# Supplementary figures and images for: The use of cultured human alveolar basal cells to mimic honeycomb formation in idiopathic pulmonary fibrosis
Source: Respir Res. 2024 Jan 10;25:26. doi: 10.1186/s12931-024-02666-9 (PMC10777517; doi:10.1186/s12931-024-02666-9)

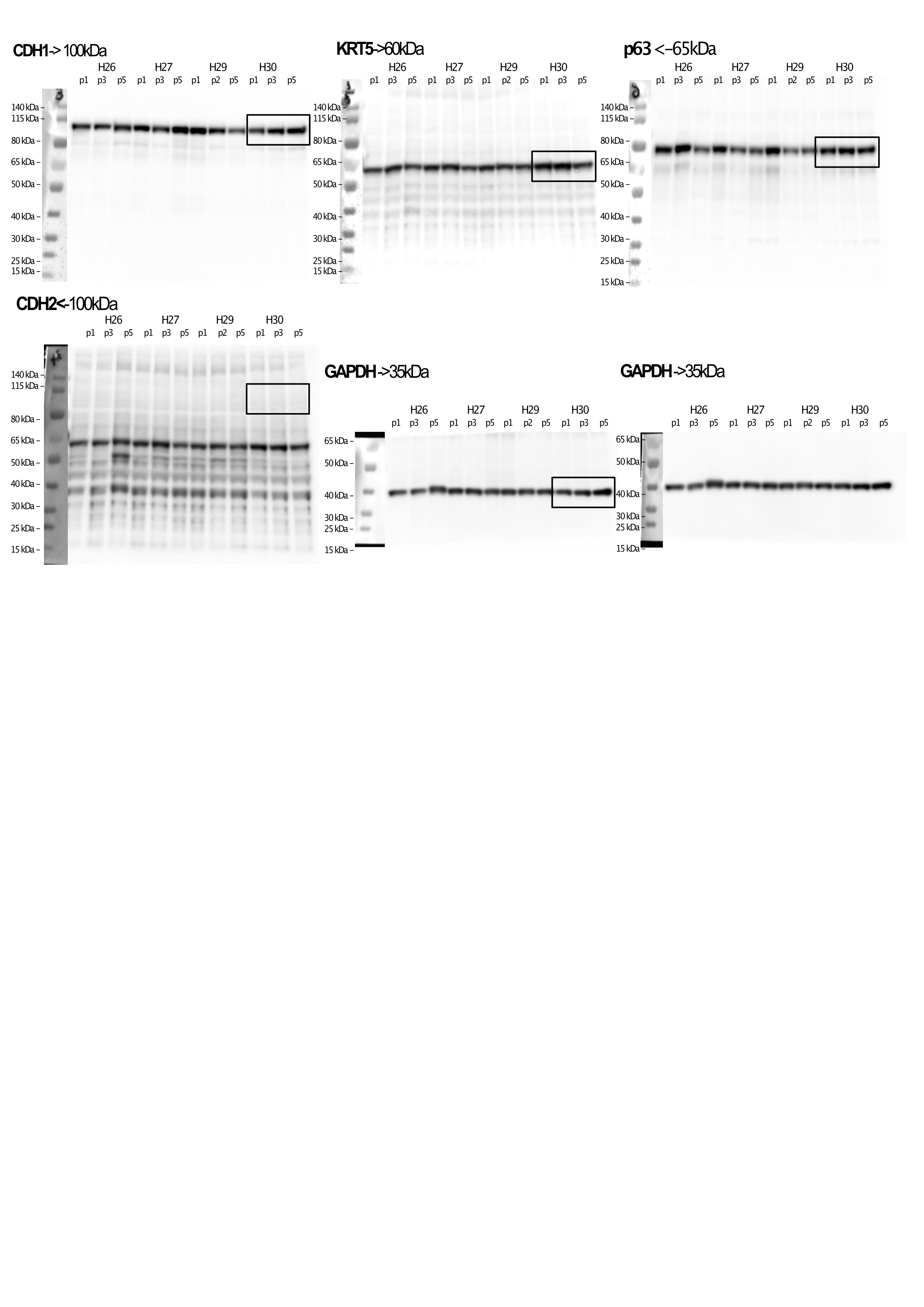

Supplement: Supplementary file 1 — Additional file 1: Figure S1. Full-length immunoblots. [file 12931_2024_2666_MOESM1_ESM.tiff]
