# Supplementary material for: The use of cultured human alveolar basal cells to mimic honeycomb formation in idiopathic pulmonary fibrosis
Source: Respir Res. 2024 Jan 10;25:26. doi: 10.1186/s12931-024-02666-9 (PMC10777517; doi:10.1186/s12931-024-02666-9)
Supplement: Supplementary file 2 — Additional file 2: Table S1. Patient characteristics. Table S2. Materials used in this study. [file 12931_2024_2666_MOESM2_ESM.pdf]

Table S1: Patient Characteristics

Patient ID	Sex	Age (years)	Clinical Diagnosis
01	Male	34	fibrosis - UIP
02	Male	60	IPF
03	Male	57	IPF
04	Male	49	fibrosis - UIP
05	Female	62	IPF
06	Male	54	IPF
07	Male	56	IPF
08	Male	60	IPF
09	Mal	54	IPF
10	Female	61	COPD/control
11	Female	74	Cancer/control
12	Female	70	Cancer/control
13	Female	61	Cancer/control

Abbreviations used: IPF, idiopathic pulmonary fibrosis; UIP, usual interstitial pneumonia.

Table S2: Materials used in this study

Product	Company	Catalog Number	Country
Cnt-PR-A	CELLnTEC	-	Bern, Switzerland
0.05% Trypsin-EDTA	Thermo Fisher Scientific	25300-054	Waltham, MA, USA
PneumaCult™-ALI medium	Stemcell Technology	05001	Cambridge, UK
Costar® 12 mm Transwell®, 0.4 μm pore polyester membrane inserts	Corning	38024	NY, USA
Heparin Solution	Stemcell Technology	07980	Cambridge, UK
Hydrocortisone stock solution	Stemcell Technology	07925	Cambridge, UK
Cultrex™ reduced growth factor basement membrane extract, type 2	RnD Systems	3533-005-02	Abingdon, UK
IMDM	Thermo Fisher Scientific	12440053	Waltham, MA, USA
F12 nutrient mix, Hams	Thermo Fisher Scientific	11765054	Waltham, MA, USA
B 27 supplement	Thermo Fisher Scientific	17504044	Waltham, MA, USA
N 2 supplement	Thermo Fisher Scientific	17502048	Waltham, MA, USA
BSA (7.5% stock)	Thermo Fisher Scientific	15260037	Waltham, MA, USA
Glutamax 100X	Thermo Fisher Scientific	35050061	Waltham, MA, USA
Ascorbic Acid (50 mg/ml stock)	Sigma Aldrich	A4544	Buchs, Switzerland
Antibiotic-antimycotic (100x)	Thermo Fisher Scientific	15240-062	Waltham, MA, USA
Monothioglycerol	Sigma Aldrich	M6145	Buchs, Switzerland
8-Bromo-cAMP	Stemcell Technology	73604	Cambridge, UK
Recombinant human FGF-2 protein	RnD Systems	233-FB-025	Abingdon, UK
Recombinant human EGF protein	PreproTech	AF-100-15	London, UK
Recombinant human FGF-10 protein	RnD Systems	345-FG-025	Abingdon, UK
A83-01	RnD Systems	2939/10	Abingdon, UK
Y-27632 dihydrochloride	RnD Systems	1254/10	Abingdon, UK
Dexamethasone	Sigma Aldrich	D4902	Buchs, Switzerland
IBMX	Stemcell Technology	72762	Cambridge, UK

Dispase II	Sigma Aldrich	D4693-1g	Buchs, Switzerland
Tissue-Tek® O.C.T. compound	Sakura	4583	Torrance, CA, USA
Mouse KRT17 antibody	Thermo Fisher Scientific	MA1-06325	Waltham, MA, USA
Rabbit KRT17 antibody	Thermo Fisher Scientific	PA5-27949	Waltham, MA, USA
Mouse KRT5 antibody	Thermo Fisher Scientific	MA5-12596	Waltham, MA, USA
Rabbit proSP-C antibody	Merck Millipore	AB3786	Darmstadt, Germany
Mouse anti-tubulin, acetylated antibody	Merck Millipore	T6793	Darmstadt, Germany
Rabbit MMP-7 antibody	Thermo Fisher Scientific	PA5-87486	Waltham, MA, USA
Rat SCGB1A1 antibody	RnD System	394324	Abingdon, UK
Goat p63 antibody	RnD System	AF1916	Abingdon, UK
Rabbit p63 antibody	Abcam	ab124762	Cambridge, MA, USA
Mouse N-cadherin (CDH2) antibody	Thermo Fisher Scientific	33-3900	Waltham, MA, USA
Rabbit E-cadherin (CDH1) antibody	Abcam	Ab40772	Cambridge, MA, USA
Rabbit GAPDH antibody	Abcam	Ab128915	Cambridge, MA, USA
Mouse MUC5AC antibody	Thermo Fisher Scientific	MA5-12178	Waltham, MA, USA
Mouse KRT14 antibody	Thermo Fisher Scientific	MA5-11599	Waltham, MA, USA
Rabbit HNA antibody	Abcam	Ab86129	Cambridge, MA, USA
Hematoxyline Erythrosin (H&E)	RAL Diagnostics	-	Bordeaux, France
TTF-1 antibody	Ventana	790-4398	Tucson, AZ, USA
Rabbit MUC5B antibody	Merck Millipore	HPA008246	Darmstadt, Germany
Alexa 647 Goat anti-mouse	Thermo Fisher Scientific	A21235	Waltham, MA, USA
Alexa 488 Donkey anti-mouse	Thermo Fisher Scientific	A21202	Waltham, MA, USA
Alexa 488 Donkey anti-rat	Thermo Fisher Scientific	A21208	Waltham, MA, USA
Alexa 488 Goat anti-rabbit	Thermo Fisher Scientific	A11008	Waltham, MA, USA
Alexa 555 Goat anti-rabbit	Thermo Fisher Scientific	A21428	Waltham, MA, USA
Alexa 594 Donkey anti-goat	Thermo Fisher Scientific	A11058	Waltham, MA, USA
Alexa 647 Goat anti-rat	Thermo Fisher Scientific	A21247	Waltham, MA, USA

DAPI	Thermo Fisher Scientific	62248	Waltham, MA, USA
Donkey anti-goat Peroxidase antibody	Sigma Aldrich	SAB3700284	Buchs, Switzerland
Goat anti-rabbit Peroxidase antibody	Sigma Aldrich	A9169	Buchs, Switzerland
Goat anti-mouse Peroxidase antibody	Sigma Aldrich	A9917	Buchs, Switzerland
Nikon Ti2-E widefield microscope	Nikon	-	-
Nikon Ti2 2.3.PO widefield microscope	Nikon	-	-
Nikon Ni – Slide scanner	Nikon	-	-
KRT5 TaqMan® Gene Expression Assay	Thermo Fisher Scientific	Hs00361185_m1	Waltham, MA, USA
KRT17 TaqMan® Gene Expression Assay	Thermo Fisher Scientific	Hs00356958_m1	Waltham, MA, USA
GAPDH TaqMan® Gene Expression Assay	Thermo Fisher Scientific	Hs03929097_g1	Waltham, MA, USA
TP63 TaqMan® Gene Expression Assay	Thermo Fisher Scientific	Hs00978340_m1	Waltham, MA, USA
KRT14 TaqMan® Gene Expression Assay	Thermo Fisher Scientific	Hs00265033_m1	Waltham, MA, USA
MUC5AC TaqMan® Gene Expression Assay	Thermo Fisher Scientific	Hs01365616_m1	Waltham, MA, USA
FOXJ1 TaqMan® Gene Expression Assay	Thermo Fisher Scientific	Hs00230964_m1	Waltham, MA, USA
SCGB1A1 TaqMan® Gene Expression Assay	Thermo Fisher Scientific	Hs00171092_m1	Waltham, MA, USA
CDH1 TaqMan® Gene Expression Assay	Thermo Fisher Scientific	Hs01023894	Waltham, MA, USA
CDH2 TaqMan® Gene Expression Assay	Thermo Fisher Scientific	Hs00983056_m1	Waltham, MA, USA
MUC5B TaqMan® Gene Expression Assay	Thermo Fisher Scientific	Hs06629268_s1	Waltham, MA, USA
TaqManTM Universal PCR Master Mix, no AmpEraseTM UNG	Thermo Fisher Scientific	4324018	Waltham, MA, USA
Quick-RNA MiniPrep Kit	ZymoResearch	R1050	Orange, CA, USA
XenoLight D-Luciferin-K+ Salt Bioluminescent Substrate	PerkinElmer	122799	USA
IVIS Lumina II	PerkinElmer	-	-
LivingImage 4.5	PerkinElmer	-	-

NucBlue Live Cell Stain Ready Probes	Thermo Fisher Scientific	R37605	Waltham, MA, USA
